# Attitudes, knowledge and practices concerning delirium among paediatric intensive care unit nurses: a multisite cross-sectional study in Sichuan, China

**DOI:** 10.1186/s12912-024-01956-3

**Published:** 2024-04-29

**Authors:** YueEr Zhang, JingYing Xie, MengLin Tang

**Affiliations:** 1 Department of Pain Management, West China Hospital ,Sichuan University/West China School of Nursing, Sichuan University, Chengdu, 610041 China; 2 West China Hospital of Stomatology,Sichuan University, Chengdu, 610041 China; 3 Department of cardiovascular surgery,West China Hospital,Sichuan University/West China School of Nursing, Sichuan University, Chengdu, 610041 China

**Keywords:** Paediatric delirium, Nurses, Knowledge-attitude-practice (KAP), Risk factors

## Abstract

**Background:**

Delirium is an acute mental state associated with poor outcomes. The incidence of delirium is high, especially in the paediatric intensive care unit (PICU). It is important for staff, particularly nurses, to understand delirium and implement interventions to prevent it. We performed a survey with the aim of evaluating and analysing the factors influencing the knowledge, attitudes and behaviour of PICU nurses towards delirium.

**Methods:**

This cross-sectional descriptive study included 215 PICU nurses in 6 PICUs from five teaching hospitals in Sichuan Province, China. An online survey about the knowledge, attitudes and practices related to delirium care was conducted among PICU nurses used a self-made and validated questionnaire. The data were analysed using descriptive statistics; differences between groups were compared using t tests, ANOVA and rank-sum tests. Variables with a significance level of 0.05 in the univariate analysis were entered into the multivariable regression analysis to identify predictors.

**Results:**

Only 14.4% of the nurses had a good understanding of delirium, and 40.9% had received relevant training. The mean knowledge score was 9.01 ± 3.86, and the overall passing rate of knowledge was 49.8%. The mean attitude and behaviour scores were 40.95 ± 5.62 and 40.33 ± 8.01, respectively. Among the hospitals, different delirium assessments for children and specific training were performed, explaining approximately 10% of the variability in knowledge scores (F = 6.152), approximately 10% of the variability in attitude/belief scores (F = 5.908), and approximately 17% of the variability in practice scores (F = 10.767).

**Conclusions:**

PICU nurses have poor knowledge of delirium, particularly regarding its clinical manifestations, influencing factors and medications used, and they have adequate attitudes and confidence and good behaviour regarding delirium in children. To better prevent delirium, we suggest that PICU departments routinely assess delirium and conduct delirium training for nurses.

**Trial registration:**

Not applicable.

**Supplementary Information:**

The online version contains supplementary material available at 10.1186/s12912-024-01956-3.

## Background

Delirium is a syndrome characterized by the acute onset of cerebral dysfunction accompanied by a change or fluctuation in baseline mental status, inattention, or either disorganized thinking or an altered level of consciousness [1]. Delirium can be classified into four clinical subtypes: hyperactive, hypoactive, mixed and unclassifiable [2]. The incidence of delirium in hospitalized children ranges from 4.5 to 65.9%, and the incidence of delirium ranges from 22 to 44% in the paediatric intensive care unit (PICU) [3, 4]. A diagnosis of delirium adds to the cost of caring for critically ill children and places them at risk for adverse outcomes, including mortality and morbidity [5]. Critically ill children with delirium experience prolonged mechanical ventilation and may experience posttraumatic stress disorder (PTSD) after hospitalization [6, 7], which means that delirium exacerbates the condition of children, prolongs hospitalization time and increases hospitalization costs. In addition, children with delirium require resource-intensive care, further increasing hospitalization costs. One study showed that delirium increases the length of hospital stay and duration of ventilation in paediatric intensive care units (PICUs) by 2–3 days and increases the cost of hospitalization by more than 4 times [8].

Early detection and aggressive treatment can reverse delirium [9], and guidelines, including assessment, identification, prevention and treatment [10], have recommended effective management of delirium in children. Nurses in intensive care units play an important role in delirium treatment; nurses have an innate advantage in the early recognition and prevention of delirium because they care for children at the bedside for long periods and provide knowledgeable care. In addition, the environment in the intensive care units(ICU) is an important factor contributing to delirium incidence, and nurses who regularly assess the impact of environmental factors and take steps to minimize the impact of these factors may reduce delirium in patients and improve outcomes. In addition, nurses play a role in educating children’s family members [11]. Delirium management should be a routine task in the PICU, but a survey showed that delirium management in the PICU is not sufficient and varies widely, which may be related to a lack of awareness of the importance of delirium among professionals, a lack of delirium-related knowledge, low confidence in the use of delirium screening tools, and fear of adverse events [12–14]. It is necessary to explore nurses’ abilities to manage delirium and to make delirium management a routine and effective task.

Knowledge-attitude-practice (KAP) theory states that targeted groups acquire relevant health or professional knowledge, form strong beliefs, and subsequently adopt positive attitudes to change their behaviours [15]. Researchers often use KAP theory as a guide to develop questionnaires to understand the knowledge, attitudes and beliefs of a population in a certain area and use the results to develop interventions to improve behaviour. Studies have been conducted to investigate delirium knowledge, attitudes and practices among health care workers, but recent investigations in this area have focused on adult ICU nurses [12, 14], with a lack of investigations involving PICU nurses. Gesin et al. [13] investigated only the knowledge of delirium among PICU nurses without evaluating their beliefs and behaviours or influencing factors.

The aims of the survey were to (1) evaluate the knowledge, attitudes and behaviour of PICU nurses regarding delirium in Sichuan Province and (2) analyse the influential factors affecting the knowledge, beliefs and behaviour of PICU nurses regarding delirium.

## Methods

### Design

A convenience sampling method was used to select PICU nurses from five large teaching hospitals (West-China Hospital of Sichuan University, Nanchong Central Hospital, Chengdu Women’s and Children’s Hospital, Second Affiliated Hospital of West China Hospital of Sichuan University, and Sichuan Women’s and Children’s Hospital) from November 2021 to January 2022 as survey respondents. The inclusion criteria were as follows: (1) registered nurses and (2) nurses working in the PICU for ≥ 3 months. The exclusion criteria were as follows: (1) nurses on leave or interns and (2) nurses who were unwilling to participate in this survey.

### Sample size

In this study, a brief calculation method was adopted, and the number of independent variables was expected to be 9; this value was calculated according to the requirement that the required sample size be 5–10 times the number of independent variables. Considering 20% attrition, the sample size should be at least 54–108 participants [16].

### Development and content of the survey

The KAP model divides the relevant behaviours into three main parts: “acquiring knowledge”, “generating beliefs”, and “forming behaviours”. The three components are continuous in time, and the results of each component are progressive. Based on this theoretical framework and the previously published ICU delirium-related questionnaire [17–19], the study questionnaire(Supplementary Appendix 1) consisted of four parts: (1) demographic and delirium training information: sex, age, education level, title, length of time in the PICU, and delirium training information; and (2) the Paediatric Delirium Knowledge Questionnaire, which consisted of 20 multiple-choice questions, including basic concepts of delirium, clinical manifestations of paediatric delirium, assessment tools, risk factors, and prevention and treatment options. Each question had three answer options (right/wrong/unclear), and each correct answer received a score of 1, while incorrect or unclear answers received a score of 0. The total possible score was 20 points. The higher the score was, the more PICU nurses knew about paediatric delirium. (3) Attitudes towards dealing with paediatric delirium: To investigate the confidence and attitudes of PICU nurses regarding intervention treatment and medical care cooperation for paediatric delirium patients, we designed 10 questions and used a 5-point Likert scale. The answer options strongly agree, agree, uncertain, disagree and strongly disagree were assigned values of 5, 4, 3, 2 and 1, respectively. The higher the score was, the greater the confidence and attitudes of nurses towards dealing with paediatric delirium. (4) Practices/Behaviour in dealing with paediatric delirium: To investigate the behaviour of PICU nurses in dealing with paediatric delirium, including prevention, intervention, medical and nursing cooperation and self-learning about delirium, a 5-point Likert scale was used, with answer options of always, often, occasionally and never (5, 4, 3, 2 and 1, respectively). With a total score ranging from 10 to 50, higher scores indicate better management behaviours of nurses in response to delirium in children.

### Validity

The content validity of the questionnaire was evaluated by experts, five experts (three PICU physicians and two nurse managers, all with a title of associate senior or above) were consulted to design the questionnaire. This questionnaire was presurveyed in 20 nurses and evaluated by 5 experts. The content validity was calculated as the proportion of experts who scored 4 points (important) or 5 points (very important) for the evaluation item. The content validity index (I-CVI) of each item was 0. 85∼1. 00, the content validity index (S-CVI) of the total scale was 0.891, and the Cronbach’s ɑ values of the three subquestionnaires were 0.907, 0.878, and 0.910, respectively.

### Ethical issues and survey methodology

The study protocol was reviewed and approved by the ethics committee of West China Hospital, Sichuan University (IRB number: 2023 − 886). All participants provided informed consent after they were informed about the study overview. An electronic survey was also conducted among the PICU nurses. Nurses could use cell phones or computers to complete the survey, and only one questionnaire could be completed and submitted for each IP address, which prevented them from filling out the questionnaires repeatedly and ensured the quality of the survey. Before sending out the link, we communicated with PICU nurse managers, explaining the purpose of this survey and obtaining permission. Then, we determined the survey time and survey person in charge. Before the nurses were distributed the electronic questionnaires, we provided uniform guidelines through the WeChat platform to explain how to answer the questionnaires in detail. The questionnaires were reviewed in a timely manner after being collected by a computer to eliminate invalid questionnaires. A total of 300 questionnaires were distributed, and 231 questionnaires were collected. After deleting invalid questionnaires, 215 valid questionnaires remained.

### Data analysis

Descriptive statistics included frequencies and percentages of demographic data. Knowledge/Attitude/Behaviour scores are expressed as the mean scores and mean percentages as previously defined. Differences between groups (e.g., knowledge scores) were compared using t tests, ANOVA and rank-sum tests. Variables with a significance level of 0.05 in the univariate analysis were subsequently entered into the multivariable regression analysis to identify predictors. All analyses were carried out under the advice of experts and performed using SPSS v 22. A *p* value < 0.05 was considered to indicate statistical significance.

## Results

### General characteristics

A total of 300 questionnaires were distributed in this study, and 215 valid questionnaires were returned, for a rate of 71.7%. Table 1 shows the general profile of the PICU nurses who participated in the survey. There were 209 (97.2%) females and 6 (2.8%) males aged 22–53 years, with an average age of 30.60 ± 5.93 years. 79% of the nurses had a bachelor’s degree. A total of 58.1% of the nurses had the title of nurse practitioner; 33.0% of the nurses had 10–20 years of experience; 36.3% of the nurses’ units did not routinely perform delirium assessment in children; and 53.5% of the nurses had cared for children with delirium. Only 40.9% of the nurses had received training in paediatric delirium, and only 14.4% felt that they had a good understanding of the topic.

**Table 1:** General characteristics and influencing Factors of delirium Knowledge-Attitude/Belief-Practice among PICU nurses (*N* = 215)

Characteristic	Group	Frequency /Percentage(%)	Knowledge		Attitude/Belief		Practice	
			Median(IQR)	Z/H	Mean ± SD	t/F	Median(IQR)	Z/H
Gender	Male	6(2.8)	8.5(5.5,11.5)	-0.464	40.33 ± 4.67	−0.275	37.5(35.25,42)	-1.028
	Female	209(97.2)	10(7,12)		40.97 ± 5.66		42(35,47)	
Age	≤ 30	116(54.0)	10(7,13)	3.541	40.92 ± 5.78	0.027	42(35,47)	0.780
	31–40	86(40.0)	9.5(6, 11.25)		41.04 ± 5.57		41.5(35,47)	
	>40	13(6.0)	9(4.5,10.5)		40.69 ± 4.87		41(32.5,45)	
Hospital	Nanchong Central Hospital	82(38.13)	11(7,13)	15.740^*^	41.75±6.20	4.538^*^	42.5(40,49)	27.889^**^
	West china hospital	58(27.0)	9.5(7,11)		41.94±4.86		43.5(34,45)	
	Second Affiliated Hospital of West China Hospital	21(9.8)	10(9,11)		40.66±4.52		39.0(34,45)	
	Chengdu Women’s and Children’s Hospital	26(12.1)	8.0(4,10)		36.92±5.23		34.5(31,39)	
	Sichuan Women’s and Children’s Hospital	28(13.0)	8.5(6,10)		40.53±4.99		40.0(31.5,45)	
Highest Level of Education	Associate degree	36(16.7)	9(7,13)	1.110	40.86 ± 7.71	0.054	41.5(30.25,48.75)	4.673
	Bachelor’s Degree	171(79.5)	10(7,12)		41.00 ± 5.29		42(36,47)	
	Master’s Degree and Phd	8(3.7)	10(6.75,13.5)		40.37 ± 5.37		34(30.75,41.75)	
Job Title	Nurse	44(20.5)	9.5(7,12)	3.386	40.54±5.88	0.762	42.5(37.25,49.75)	7.019
	Senior Nurse	125(58.1)	10(7,13)		41.37±5.76		42(36,48)	
	Supervisor Nurse and above	46(21.4)	9(6,11)		40.21±4.96		38(32.75,44.25)	
Years of work as registered nurses	<1	9(4.2)	10(9,12.5)	7.112	41.00 ± 4.41	0.081	44(35.5,50)	3.649
	1–5	66(30.7)	10(8,13)		40.84 ± 5.77		41(35.75,47.25)	
	6–10	56(26.0)	9.5(6.25,12.75)		41.30 ± 5.66		44(35.5,47)	
	10–20	71(33.0)	9(5,11)		40.85 ± 5.82		40(33,47)	
	>20	13(6.0)	9(4.5,10.5)		40.53 ± 4.96		41(32.5,43)	
Does the department perform delirium assessment?	Yes	137(63.7)	10(7.5,12.5)	-2.795^*^	41.92 ± 4.94	3.204^*^	43(39,48)	-4.580^**^
	No	78(36.3)	9(5,11)		39.25 ± 6.34		35(30,45)	
Have cared for a child with delirium	Yes	115(53.5)	9(6,11)	7.384^*^	40.86 ± 5.35	0.050	41(35,47)	0.882
	No	86(40.0)	10.5(7.75,13)		41.10 ± 5.74		42(34,49)	
	Not clear	14(6.5)	8.5(6.5,10.25)		40.78 ± 7.33		42(33.75,44.25)	
	Somewhat	157(73.0)	9(6.5,12)		40.88 ± 5.41		40(35,47)	
	Don’t know	27(12.6)	7(2,12)		37.29 ± 5.97		35(30,45)	
Received specific training	Yes	88(40.9)	10(8,13)	-3.353^**^	42.61 ± 4.39	3.915^**^	43(39,48)	-3.328^**^
	No	127(59.1)	9(6,11)		39.81 ± 6.10		39(32,46)	

*:*p*<0.05, **: *p* ≤ 0.001, IQR: Interquartile Range, Z:Z-value, H:H-value, SD: Standard Deviation, t:t-value, F: F-value

### Attitudes, knowledge and practices regarding delirium among PICU nurses

The highest score for PICU nurses’ knowledge of delirium in children was 17 points, with no nurses receiving a perfect score and only 49.8% (107/215) of the nurses scoring more than 12 points. Only 4.2% of the nurses knew that “children usually remember delirium”. In addition, the nurses’ scores regarding the factors influencing delirium, such as family history (history of dementia), clinical manifestations (duration, symptoms of hyperactivity), and medications used to treat delirium, were also very low. A total of 71.6% of the nurses believed that benzodiazepines were helpful in treating delirium. A total of 29.3% of the nurses believed that children with delirium always presented with a hyperactive, confused state. (Supplementary Appendix 2). A perfect score regarding the confidence and attitudes of nurses regarding delirium was given by 8.8% (19/215) of the nurses, and 98.6% (212/215) of the nurses received a passing score (≥ 30 points) (Supplementary Appendix 2). The three questions with the lowest mean scores are shown in Supplementary Appendix 2, where the mean score is “If asked, are you confident that you can provide an accurate definition of delirium?” was 3.32 ± 1.03, with only 16.3% of the nurses being very confident that they could provide an accurate definition of delirium. This was followed by “You are able to use at least two interventions to prevent and reduce the duration of delirium in PICU patients”, with a mean score of 3.66 ± 0.97 and 21.8% of the nurses being very confident in the use of the two interventions. In addition, the mean score for “You are confident in communicating concerns about the presence or risk of delirium to the child’s bedside physician” was 3.83 ± 0.97, with 28.4% of the nurses being very confident in communicating delirium prevention and recognition to the physician.

Of the nurses’ behaviours in response to delirium, 15.8% (34/215) received a perfect score, and 91.6% (196/215) received a passing score (≥ 30) (Table 2). The three questions with the lowest mean scores are shown in Table 3. The mean score for “encourage family companionship as much as possible when appropriate” was 3.69 ± 1.22, and only 34.4% of the nurses strongly agreed that family companionship should be encouraged. The mean score for “Actively learn about delirium in children” was 3.72 ± 1.15, with 34% of the nurses always actively learning about delirium in children. In addition, the mean score for “Frequently use ICU delirium assessment tools in clinical work” was 3.77 ± 1.36, with 41.4% of the nurses always using delirium assessment tools.

**Table 2:** Knowledge-Attitude/Belief-Practice of delirium among PICU nurses (*N* = 215)

	Score range	Mean ± SD	Passing Percentage
Knowledge	0–17	9.01 ± 3.86	49.8%
Attitude/Belief	25–50	40.95 ± 5.62	98.6%
Practice	10–50	40.33 ± 8.01	91.2%

SD: Standard Deviation

### Factors associated with PICU nurse attitudes, knowledge and practices concerning delirium

The test analysis has showed that the departments conducting delirium assessments, caring for children with delirium, ensuring an understanding of delirium, and providing delirium training affected PICU nurses’ knowledge of delirium in children; nurses in different hospitals scored differently, in addition to nurses whose departments conducted delirium assessments, cared for children with delirium, ensured an understanding of delirium information, and provided delirium training with higher delirium knowledge scores. The departments that conduct delirium assessments, ensure an understanding of delirium, and provide delirium training affect PICU nurses’ beliefs and behaviours regarding delirium in children. Nurses whose departments conducted delirium assessments, ensured an understanding of delirium information, and provided delirium training had higher delirium confidence and coping behaviour scores (Table 1). In addition, delirium behaviour scores differed among nurses with different titles, with nurses with lower titles having higher delirium behaviour scores. We subsequently included all the factors in a multiple regression analysis, which showed that not receiving specific training had the greatest impact on knowledge scores. The departments did not perform delirium assessments, and not receiving specific training had an impact on nurses’ delirium beliefs. Different hospitals and not performing delirium assessments had an impact on nurses’ delirium beliefs and behaviours (Table 3).

**Table 3:** Multivariable regression analysis of delirium Knowledge-Attitude/Belief-Practice among PICU nurses (*N* = 215)

Predictor	Knowledge	Attitude/Belief	Practice
	β		
	(95%CI)		
	*P* -Value		
Hospital	−0.103	−0.136	−0.189
	(−0.672 to 0.115)	(−0.111 to 0.003)	(−0.185 to−0.28)
	0.164	0.065	**0.008**
Does the department perform delirium assessment?	−0.151	−0.155	−0.310
	(−2.437 to 0.025)	(−1.360 to−0.289)	(−0.762 to−0.270)
	0.055	**0.021**	**0.000**
Have cared for a child with delirium	0.104	/	/
	(−0.310 to 1.605)	/	/
	0.184	/	/
Received specific training	−0.194	−0.163	−0.078
	(−2.770 to−0.266)	(−0.369 to−0.003)	(−0.377 to 0.124)
	**0.018**	**0.047**	0.320
F	6.152^**^	5.908^**^	10.767^**^
R^2^	0.105	0.101	0.170

*:*p*<0.05; **: *p* ≤ 0.001, β: β-value, 95%CI:95% Confidence lnterval, F: F-value, R^2^:R-squared

## Discussion

The most important findings of this multicentre survey on delirium among PICU nurses are summarized as follows:

1. PICU nurses have poor knowledge of delirium in children, especially regarding clinical manifestations, some influencing factors and medications used;
2. PICU nurses have adequate beliefs and confidence and good behaviour regarding delirium in children; and It is important to conduct delirium training for PICU nurses.

In this study, 49.8% of the PICU nurses received a passing score for delirium knowledge, and the delirium knowledge of the PICU nurses was poor, which has been mentioned in numerous previous studies [20, 21]. The attitude/belief and practice scores of the PICU nurses were 98.6% and 91.2%, respectively, indicating that the nurses had a certain awareness of delirium and paid attention to it. However, considering that knowledge of delirium is poor, nurses may not be able to carry out effective delirium management. Knowledge deficiencies are considered major obstacles to the implementation of delirium guidelines, and there is a need to strengthen continuing education to equip nurses with the latest knowledge of delirium care [22].

According to the items in the attitudes and practices items where PICU nurses scored low(Supplementary Appendix 2), suggested that PICU nurses were aware of the importance of delirium management but did not have the confidence to manage delirium well; for example, they could implement preventive measures and conduct delirium assessments and subsequently performed poorly on delirium management behaviours. This finding confirms those of Eastwood et al. [23] and Devlin et al [24], who reported that different ICUs assess delirium in different ways and that 85% of nurses do not use assessment tools, which may be related to nurses’ lack of understanding of delirium assessment tools and the fact that the assessment tools are complex and require additional time and effort.

Analysis of the factors affecting the delirium knowledge and belief behaviour of PICU nurses revealed that age, sex, years of experience, education level and title had no effect on the delirium knowledge or belief behaviour of nurses. In contrast, some studies have shown that nurses with low seniority and education levels have relatively low delirium knowledge [18, 21]. In addition, we explored whether the units assessed delirium had an effect on PICU nurses’ delirium knowledge and belief behaviour. KAP theory states that improving cognition improves the ability to act. Accordingly, it is necessary to establish a delirium assessment program in PICU departments to influence nurses’ understanding of the management of this disease and improve their ability to care for delirium.

Finally, training is an important and effective measure for improving the knowledge, attitudes and behaviour of PICU nurses regarding delirium. The current study showed that while nurses often care for patients with delirium, most nurses report a lack of educational experience, and there are no delirium assessment tools or manuals available in their departments [25]. However, one study showed that after nurse training, the incidence of delirium decreased by 54.29% [26]. Therefore, it is necessary to develop an education and training program that reflects the needs identified in this study so that hospital nurses can develop the knowledge, skills, and attitudes needed for delirium care.

### Limitations

This study has several limitations. First, the generalizability of our study results to other contexts is limited because we targeted hospital nurses working at five hospitals in Sichuan Province, China, by convenience sampling. Second, the questionnaire used in this study was self-designed, and although it was reviewed by experts, it was not validated by additional psychometric indices and was used on a large scale. Furthermore, the potential for selection bias should be accounted for because of the 71.7% response rate.

### Implications for practice

PICU nurses are in a unique position to care for patients at the bedside for long periods and are an important part of the early identification and prevention of delirium. In addition, due to the special environment of the PICU, the absence of family members and the weak expression abilities of children, it is necessary for nurses to improve their understanding and attitudes regarding delirium. To improve PICU nurses’ competency in delirium care, we recommend creating a good atmosphere in the department. Managers need to develop appropriate delirium management strategies, such as specifying the frequency of delirium assessment and providing assessment tools for paediatric delirium. In addition, it is necessary to develop delirium training programs to improve nurses’ knowledge and attitudes regarding delirium.

## Conclusions

The online investigation results of PICU nurses show that PICU nurses lack the knowledge and practical management ability of delirium in children, but PICU nurses have adequate beliefs and confidence and good behaviour regarding delirium in children. The training experience, caregiving experience of children with delirium, and their department’s regulations on delirium management were influence factors the knowledge, attitude/belief, and practice of PICU nurses on delirium in children. Further research is needed to determine the effects of training programs and management strategies on the improvement of the delirium care competency of hospital nurses.

### Electronic supplementary material

Below is the link to the electronic supplementary material.

Supplementary Material 1

Supplementary Material 2

## Data Availability

The data that support the findings of this study are available from the corresponding author upon reasonable request.

## Abbreviations

PICU: Paediatric Intensive Care Unit
ICU: Intensive Care Unit
KAP: Knowledge-Attitude-Practice
ANOVA: Analysis of variance
SPSS: Statistical Product and Service Solutions
IRB: Institutional Review Board
