# Supplementary material for: Attitudes, knowledge and practices concerning delirium among paediatric intensive care unit nurses: a multisite cross-sectional study in Sichuan, China
Source: BMC Nurs. 2024 Apr 29;23:289. doi: 10.1186/s12912-024-01956-3 (PMC11057075; doi:10.1186/s12912-024-01956-3)
Supplement: Supplementary file 1 — Supplementary Material 1 [file 12912_2024_1956_MOESM1_ESM.docx]

**Supplementary Appendix 1- Questionnaire Investigation of knowledge, attitude and practices of PICU nurses on delirium**

***Part one* General characteristics**

1. Gender

| ○male |
| --- |
| ○Female |

2. Age

_________________________________

3. Level of education

| ○Associate degree  ○Bachelor’s Degree  ○Master’s Degree and PhD |
| --- |

4.Job title

| ○Nurse  ○Senior Nurse  ○Supervisor Nurse and above |
| --- |

5.Years of work as a registered nurse

| ○＜1  ○1-5  ○6-10  ○10-20  ○＞20 |
| --- |

6. Workplace

_________________________________

7.Does the department perform delirium assessment?

| ○Yes |
| --- |
| ○No |

8. Have cared for a child with delirium

| ○Yes  ○No  ○Not clear  ○Somewhat  ○Do not know |
| --- |

9.Have received specific training about delirium

| ○Yes  ○No |
| --- |

***Part two*** **Paediatric Delirium Knowledge Questionnaire**

1.The typical features of delirium do not include fluctuations between disorientation and disorientation

| ○ True  ○ False  ○ Not clear |
| --- |

2.Behavioral changes throughout the day are typical manifestations of delirium

| ○ True |
| --- |
| ○ False |
| ○ Not clear |

3.Delirious patients often have perceptual difficulties

| ○ True |
| --- |
| ○ False |
| ○ Not clear |

4.An altered sleep/wake cycle may be a symptom of delirium

| ○ True |
| --- |
| ○ False |
| ○ Not clear |

5.Delirium usually lasts for hours

| ○ True |
| --- |
| ○ False |
| ○ Not clear |

6.The symptoms of delirium can be similar to those of depression

| ○ True |
| --- |
| ○ False |
| ○ Not clear |

7.The Glasgow score is the best way to diagnose delirium in PICU children

| ○ True |
| --- |
| ○ False |
| ○ Not clear |

8.Impairment of hearing or vision can increase the risk of delirium

| ○ True |
| --- |
| ○ False |
| ○ Not clear |

9.Delirium in children is always characterized by hyperactivity and confusion

| ○ True |
| --- |
| ○ False |
| ○ Not clear |

10.Benzodiazepines help prevent delirium

| ○ True |
| --- |
| ○ False |
| ○ Not clear |

11Malnutrition increases the risk of delirium

| ○ True |
| --- |
| ○ False |
| ○ Not clear |

12.A family history of dementia predisposes patients to delirium

| ○ True |
| --- |
| ○ False |
| ○ Not clear |

13.Preschool children are more likely to develop delirium

| ○ True |
| --- |
| ○ False |
| ○ Not clear |

14.Gender had no effect on the development of delirium

| ○ True |
| --- |
| ○ False |
| ○ Not clear |

15.Dehydration may be a risk factor for delirium

| ○ True |
| --- |
| ○ False |
| ○ Not clear |

16.The more medications a child is given, the greater their risk of developing delirium

| ○ True |
| --- |
| ○ False |
| ○ Not clear |

17.Catheterization can reduce the risk of delirium

| ○ True |
| --- |
| ○ False |
| ○ Not clear |

18.Children usually do not experience delirium memories

| ○ True |
| --- |
| ○ False |
| ○ Not clear |

19.Atypical antipsychotics such as risperidone and olanzapine may be used to control the symptoms of delirium in children

| ○ True |
| --- |
| ○ False |
| ○ Not clear |

20.Patients who are comatose for most of the day do not screen positive for delirium

| ○ True |
| --- |
| ○ False |
| ○ Not clear |

***Part three* Attitudes towards dealing with paediatric delirium**

1.It is important to evaluate children for delirium

| ○Can't agree more |
| --- |
| ○ Agree |
| ○ Normal |
| ○ Disagreement  ○ Strongly disagree |

2.If asked, you are confident that you can provide an accurate definition of delirium

○Can't agree more

○ Agree

○ Normal

○ Disagreement

○ Strongly disagree

3.You are confident that you will communicate concerns about the presence or risk of delirium to the child's tube bed doctor

○Can't agree more

○ Agree

○ Normal

○ Disagreement

○ Strongly disagree

4.There are at least two interventions you can use to prevent and reduce delirium in children with PICU

○Can't agree more

○ Agree

○ Normal

○ Disagreement

○ Strongly disagree

5.You believe that daily assessment of delirium in children with PICU is a worthwhile intervention

| ○Can't agree more  ○ Agree  ○ Normal  ○ Disagreement  ○ Strongly disagree |
| --- |

6.You think it is necessary to set up a special person to manage delirium in children

○Can't agree more

○ Agree

○ Normal

○ Disagreement

○ Strongly disagree

1. You believe that nursing is very important for the prevention and prognosis of delirium in children

○Can't agree more

○ Agree

○ Normal

○ Disagreement

○ Strongly disagree

8.You think the nurse should be responsible for the identification of delirium

○Can't agree more

○ Agree

○ Normal

○ Disagreement

○ Strongly disagree

9.You believe that delirium has a serious impact on the prognosis of the child

○Can't agree more

○ Agree

○ Normal

○ Disagreement

○ Strongly disagree

10. You think that the occurrence of delirium in children is not conducive to medical quality and safety

○Can't agree more

○ Agree

○ Normal

○ Disagreement

○ Strongly disagree

***Part four* Behaviour in dealing with paediatrics delirium**

1.Alert the doctor/nurse to the child's mental/conscious state

○ All the time

○ Often

○ Sometimes

○ Occasionally

○ Never

2.Minimize light, sound and other stimuli to children at night; Avoid sleep deprivation with noise cancellation, eye masks, or earplugs

○ All the time

○ Often

○ Sometimes

○ Occasionally

○ Never

3.The sedated children were given daily arousal (or daily sedation interruption) and cognitive stimulation

○ All the time

○ Often

○ Sometimes

○ Occasionally

○ Never

4.Encourage family members as much as is appropriate

○ All the time

○ Often

○ Sometimes

○ Occasionally

○ Never

5.To assist children in early recovery when the condition permits

○ All the time

○ Often

○ Sometimes

○ Occasionally

○ Never

6.You frequently use the ICU delirium assessment tool in your clinical work

○ All the time

○ Often

○ Sometimes

○ Occasionally

○ Never

7.When the child develops delirium, you actively cooperate with the doctor

○ All the time

○ Often

○ Sometimes

○ Occasionally

○ Never

8.If a child develops delirium, you will pay close attention to the treatment effect

○ All the time

○ Often

○ Sometimes

○ Occasionally

○ Never

9.You follow delirium-related guidelines for clinical care practice and build knowledge in your work

○ All the time

○ Often

○ Sometimes

○ Occasionally

○ Never

10.You will take the initiative to learn about delirium in children

○ All the time

○ Often

○ Sometimes

○ Occasionally

○ Never
