# Supplementary material for: Attitudes, knowledge and practices concerning delirium among paediatric intensive care unit nurses: a multisite cross-sectional study in Sichuan, China
Source: BMC Nurs. 2024 Apr 29;23:289. doi: 10.1186/s12912-024-01956-3 (PMC11057075; doi:10.1186/s12912-024-01956-3)
Supplement: Supplementary file 2 — Supplementary Material 2 [file 12912_2024_1956_MOESM2_ESM.doc]

supplementary material-2

knowledge, attitudes and practice questionnaire correct rate/score of each item among PICU nurses(N=215)

| **Item** | **Correct rate(%)/**  **average score(Mean± SD)** | |
| --- | --- | --- |
| **Knowledge** | |  |
| Children generally don’t remember being delirious (FALSE) | | 9(4.2%) |
| A family history of dementia predisposes a patient to delirium (FALSE) | | 17(8.0%) |
| Delirium usually lasts several hours (FALSE) | | 22(10.2%) |
| Benzodiazepines help prevent delirium(FALSE) | | 61(28.3%) |
| Delirium in children is always characterized by hyperactivity and confusion(FALSE) | | 63(29.3%) |
| Patients who are comatose for most of the day do not screen positive for delirium(FALSE) | | 69(32.1%) |
| Gender had no effect on the development of delirium (FALSE) | | 80(37.2%) |
| Preschool children are more likely to develop delirium(TRUE) | | 82(38.1%) |
| The symptoms of delirium can be similar to those of depression(TRUE) | | 94(43.7%) |
| The typical features of delirium do not include fluctuations between disorientation and disorientation(FALSE) | | 95(44.2%) |
| The Glasgow score is the best way to diagnose delirium in PICU children(FALSE) | | 95(44.2%) |
| Behavioral changes throughout the day are typical manifestations of delirium(TRUE) | | 108(50.2%) |
| Malnutrition increases the risk of delirium(TRUE) | | 129(60.0%) |
| Dehydration may be a risk factor for delirium(TRUE) | | 129(60.0%) |
| Atypical antipsychotics such as risperidone and olanzapine may be used to control the symptoms of delirium in children(TRUE) | | 133(62%) |
| Catheterization can reduce the risk of delirium(FALSE) | | 135(63.0%) |
| The more medications a child is given, the greater their risk of developing delirium(TRUE) | | 145(67.4%) |
| Delirious patients often have perceptual difficulties(TRUE) | | 146(67.9%) |
| Impairment of hearing or vision can increase the risk of delirium(TRUE) | | 149(69.3%) |
| An altered sleep/wake cycle may be a symptom of delirium(TRUE) | | 177(82.3%) |
| **Attitude/Belief** | |  |
| If asked, are you confident that you can provide an accurate definition of delirium | | 3.32±1.03 |
| There are at least two interventions you can use to prevent and reduce the time to delirium in PICU | | 3.66±0.97 |
| You are confident that you can communicate concerns about the presence or risk of delirium to doctor | | 3.83±0.97 |
| You think the nurse should be responsible for the identification of delirium | | 4.15±0.857 |
| You believe that daily assessment of delirium in children with PICU is a worthwhile intervention | | 4.24±0.764 |
| You think it is necessary to set up a special person to manage delirium in children | | 4.21±0.756 |
| You believe that delirium has a serious impact on the prognosis of the child | | 4.20±0.748 |
| You think that the occurrence of delirium in children is not conducive to medical quality and safety | | 4.31±0.729 |
| You believe that nursing is very important for the prevention and prognosis of delirium in children | | 4.42±0.636 |
| It is important to evaluate children for delirium | | 4.62±0.549 |
| **Practice** | |  |
| You can encourage family companionship as much as possible if appropriate | | 3.69±1.22 |
| You take the initiative to learn about delirium in children in general | | 3.72±1.15 |
| You frequently use the ICU Delirium Assessment Tool in your clinical work | | 3.77±1.36 |
| The sedated children were given daily arousal (or daily sedation interruption) and cognitive stimulation | | 3.85±1.138 |
| You follow delirium-related guidelines for clinical care practice and build knowledge in your work | | 4.01±1.121 |
| You frequently use the ICU delirium assessment tool in your clinical work | | 4.26±1.003 |
| Alert the doctor/nurse to the child's mental/conscious state | | 4.25±0.995 |
| If a child develops delirium, you will pay close attention to the treatment effect | | 4.26±0.964 |
| Minimize light, sound and other stimuli to children at night; Avoid sleep deprivation with noise cancellation, eye masks, or earplugs | | 4.23±0.887 |
| To assist children in early recovery when the condition permits | | 4.3±0.824 |

SD:Standard Deviation
